# Supplementary material for: Privacy nudges for disclosure of personal information: A systematic literature review and meta-analysis
Source: PLoS One. 2021 Aug 27;16(8):e0256822. doi: 10.1371/journal.pone.0256822 (PMC8396794; doi:10.1371/journal.pone.0256822)
Supplement: S1 Appendix — (DOCX) [file pone.0256822.s002.docx]

**S1 Appendix**

**Search strategy**

Since each database has different requirements, we adapted the search terms per database in order to produce the most efficient results. As a result, in Scopus, Web of Science and Science Direct all search terms depicted on Table 1 were used, searching in the abstract of papers. However, the terms were adapted for Google Scholar and ACM Digital Library as below:

- Google Scholar

(“privacy”) AND (“personal information”|” personal data”) AND ("disclosure"|'sharing") AND ("nudge"|"nudging"|"choice architecture")

- ACM Digital Library

+“privacy”+( “personal information” “personal data” “sensitive information”)+( “disclosure” “share”)+(“nudge” “nudging” “experiment”)

**Quality Assessment**

The questions were scored as follows:

- QA1: Y (yes), the aim of the study is clearly defined; N (no) the aim of the study has not been described and cannot be inferred.
- QA2: Y, the study describes adequately the context of the research; N the context of the study is not clearly defined and cannot be inferred.
- QA3: Y, the research design of the study is appropriate to address its aims; N, research design of the study is not appropriate to address the aims of the research.
- QA4: Y, the study includes a control or comparison group; N, the study does not include a control group.
- QA5: Y, data collection methods are adequately described in the study; N, the methods that were used in order to collect data were not described explicitly.
- QA6: the authors of the study explicitly mention the source or the development process of the measure; N, measures are not defined, and authors do not mention the source or development of the instrument.
- QA7: Y, the experimental design is appropriate for the purposes of the study; N, the experimental design is not suitable to address the aims of the study.
- QA8: Y, statistical methods are explicitly described in the study and the purpose of the analysis is clearly defined in the paper; N, statistical methods are not explicitly mentioned and cannot be inferred, and the purpose of the analysis has not been described and cannot be inferred.
- QA9: Y, findings of the study have been clearly stated and discussed; N, results have been partially mentioned or not stated at all.
- QA10: Y, results contribute both to the academia and practice; N, the results of the study contribute neither to academia nor practice with a minimum impact.

**Table A1. Quality Assessment**

| **Author** | **Year** | **Title** | **QA Score** |
| --- | --- | --- | --- |
| Acquisti, A., John, L. K., & Loewenstein, G. | 2012 | The Impact of Relative Standards on the Propensity to Disclose | 10 |
| Adjerid, I., Acquisti, A., Brandimarte, L., & Loewenstein, G | 2013 | Sleights of Privacy: Framing, Disclosures, and the Limits of Transparency | 10 |
| Adjerid, I., Peer, E., & Acquisti, A | 2018 | Beyond the Privacy Paradox: Objective versus Relative Risk in Privacy Decision Making | 10 |
| Aiken, K. D., & Boush, D. M | 2006 | Trustmarks, objective-source ratings, and implied investments in advertising: Investigating online trust and the context-specific nature of internet signals | 10 |
| Alemany, J., del Val, E., Alberola, J. and García-Fornes, A. | 2019 | Enhancing the privacy risk awareness of teenagers in online social networks through soft-paternalism mechanisms | 9 |
| Babula, E., Mrzygłód, U., & Poszewiecki, A | 2017 | Consumers’ need of privacy protection – Experimental results | 10 |
| Baek, Y. M., Bae, Y., Jeong, I., Kim, E., & Rhee, J. W | 2014 | Changing the default setting for information privacy protection: What and whose personal information can be better protected? | 10 |
| Balebako, R., Péer, E., Brandimarte, L., Cranor, L., & Acquisti, A | 2013 | Is it the Typeset or the Type of Statistics? Disfluent Font does not Reduce Self-disclosure | 10 |
| Becker, M., Matt, C. and Hess, T. | 2020 | It's not just about the product: How persuasive communication affects the disclosure of personal health information | 10 |
| Ben-shahar, O., & Chilton, A | 2016 | Simplification of privacy disclosures: an experimental test. | 10 |
| Betzing, J. H., Tietz, M., vom Brocke, J. and Becker, J. | 2020 | The impact of transparency on mobile privacy decision making | 10 |
| Bhatia, J., Breaux, T. D., Reidenberg, J. R., & Norton, T. B | 2016 | A Theory of Vagueness and Privacy Risk Perception | 8 |
| Brandimarte, L., Acquisti, A., & Loewenstein, G | 2013 | Misplaced Confidences: Privacy and the Control Paradox | 9 |
| Carpenter, S., Shreeves, M., Brown, P., Zhu, F., & Zeng, M | 2018 | Designing Warnings to Reduce Identity Disclosure | 10 |
| Carpenter, S., Zhu, F., & Kolimi, S | 2014 | Reducing online identity disclosure using warnings. | 10 |
| Carpenter, S., Zhu, F., Zeng, M., & Shreeves, M | 2017 | Expert Sources in Warnings May Reduce the Extent of Identity Disclosure in Cyber Contexts | 10 |
| Chang, D., Krupka, E. L., Adar, E., & Acquisti, A | 2016 | Engineering Information Disclosure: Norm Shaping Designs | 10 |
| Craciun, G. | 2018 | Choice defaults and social consensus effects on online information sharing: The moderating role of regulatory focus | 9 |
| Eling, N., Rasthofer, S., Kolhagen, M., Bodden, E., & Buxmann, P | 2016 | Investigating users’ reaction to fine-grained data requests: A market experiment | 10 |
| Feri, F., Giannetti, C., & Jentzsch, N | 2016 | Disclosure of personal information under risk of privacy shocks. | 10 |
| Frey, R. M., Bühler, P., Gerdes, A., Hardjono, T., Fuchs, K. L., & Ilic, A | 2017 | The Effect of a Blockchain-Supported, Privacy- Preserving System on Disclosure of Personal Data | 10 |
| Gabisch, J. A., & Milne, G. R. | 2013 | Self-disclosure on the web: Rewards, safety cues, and the moderating role of regulatory focus | 10 |
| Gerlach, J., Widjaja, T., & Buxmann, P. | 2015 | Handle with care: How online social network providers’ privacy policies impact users’ information sharing behavior | 10 |
| Halevi, T., Kuppusamy, T. K., Caiazzo, M., & Memon, N | 2015 | Investigating users’ readiness to trade-off biometric fingerprint data. | 9 |
| Hanson, J., Wei, M., Veys, S., Kugler, M., Strahilevitz, L. and Ur, B. | 2020 | Taking Data Out of Context to Hyper-Personalize Ads | 10 |
| Huang, N., Hong, Y., Chen, P.-Y., & Wu, S.-Y. | 2018 | Digital Nudging for Online Social Sharing: Evidence from A Randomized Field Experiment | 10 |
| Hughes-roberts, T. | 2015 | Privacy as a secondary goal problem: an experiment examining control. | 10 |
| Hui, K., Teo, H., & Lee, S | 2007 | The Value of Privacy Assurance: An Exploratory Field Experiment. | 9 |
| Hutton, L., Henderson, T., & Kapadia, A. | 2014 | Here I am, now pay me!”: Privacy concerns in incentivised location-sharing systems. | 9 |
| Ilany Tzur, N., Zalmanson, L., & Oestreicher-Singer, G | 2016 | The Dark Side of User Participation - The Effect of Calls to Action on Trust and Information Revelation | 10 |
| John, L. K., Acquisti, A., & Loewenstein, G. | 2011 | Strangers on a Plane: Context-Dependent Willingness to Divulge Sensitive Information | 10 |
| Junger, M., Montoya, L., & Overink, F. J. | 2017 | Priming and warnings are not effective to prevent social engineering attacks. | 10 |
| Keith, M. J., Fredericksen, J. T., Reeves, K. S., & Babb, J | 2018 | Optimizing Privacy Policy Videos to Mitigate the Privacy Policy Paradox. | 10 |
| Kim, J., Gambino, A., Sundar, S., Rosson, M., Aritajati, C., Ge, J., & Fanning, C. | 2018 | Interface Cues to Promote Disclosure and Build Community: An Experimental Test of Crowd and Connectivity Cues in an Online Sexual Health Forum. | 10 |
| Knijnenburg, B. P., & Kobsa, A | 2013 | Making decisions about privacy: Information disclosure in context-aware recommender systems | 10 |
| Knijnenburg, B. P., Kobsa, A., & Jin, H | 2013 | Counteracting the negative effect of form auto-completion on the privacy calculus | 10 |
| Knijnenburg, B. P., Kobsa, A., & Jin, H. | 2013 | Preference-based Location Sharing: Are More Privacy Options Really Better? | 10 |
| Knijnenburg, B., & Kobsa, A | 2016 | Increasing sharing tendency without reducing satisfaction: finding the best privacy-settings user interface for social networks | 9 |
| Krol, K., & Preibusch, S | 2016 | Control versus Effort in Privacy Warnings for Webforms. | 10 |
| Kroschke, M., & Steiner, M | 2017 | The Influence of Social Cues on Users’ Information Disclosure Intentions – The Case of Mobile Apps | 10 |
| Lai, Y.-L., & Hui, K.-L | 2006 | Internet opt-in and opt-out. | 9 |
| Larose, R., & Rifon, N. | 2007 | Promoting i-Safety: Effects of Privacy Warnings and Privacy Seals on Risk Assessment and Online Privacy Behavior | 10 |
| Lee, D., Larose, R. | 2011 | The Impact of Personalized Social Cues of Immediacy on Consumers’ Information Disclosure | 10 |
| Li, H., Sarathy, R., & Xu, H | 2010 | Understanding situational online information disclosure as a privacy calculus. | 10 |
| Lu, Y., Ou, C. X. J., & Angelopoulos, S | 2018 | Exploring the effect of monetary incentives on user behavior in Online Sharing Platforms | 10 |
| Mamonov, S., & Benbunan-Fich, R | 2018 | The impact of information security threat awareness on privacy-protective behaviors | 10 |
| Marreiros, H., Tonin, M., Vlassopoulos, M., & Schraefel, M. C | 2017 | “Now that you mention it”: A survey experiment on information, inattention and online privacy | 10 |
| Meier, Y., Schäwel, J., Kyewski, E. and Krämer, N. C. | 2020 | Applying Protection Motivation Theory to Predict Facebook Users' Withdrawal and Disclosure Intentions | 10 |
| Mettler, T., & Winter, R. | 2016 | Are business users social? A design experiment exploring information sharing in enterprise social systems | 10 |
| Molina, M. D., Shyam Sundar, S. and Gambino, A. | 2019 | Online privacy in public places: How do location, terms and conditions and VPN influence disclosure? | 9 |
| Monteleone, S., Bavel, R. Van, Rodríguez-Priego, N., & Esposito, G | 2015 | Nudges to Privacy Behaviour: Exploring an Alternative Approach to Privacy Notices. | 10 |
| Mothersbaugh, D. L., Foxx, W. K., Beatty, S. E., & Wang, S | 2012 | Disclosure Antecedents in an Online Service Context: The Role of Sensitivity of Information. | 10 |
| Mukherjee, S., Manjaly, J. A., & Nargundkar, M | 2013 | Money makes you reveal more: Consequences of monetary cues on preferential disclosure of personal information | 10 |
| Nosko, A., Wood, E., Kenney, M., Archer, K., De Pasquale, D., Molema, S., & Zivcakova, L. | 2012 | Examining priming and gender as a means to reduce risk in a social networking context: Can stories change disclosure and privacy setting use when personal profiles are constructed? | 10 |
| Peer, E., & Acquisti, A | 2016 | The impact of reversibility on the decision to disclose personal information. | 10 |
| Preibusch, S., Krol, K., & Beresford, A. R | 2013 | The Privacy Economics of Voluntary Over-disclosure in Web Forms. | 9 |
| Premazzi, K., Castaldo, S., Grosso, M., Raman, P., Brudvig, S., & Hofacker, C. F. | 2010 | Customer Information Sharing with E-Vendors: The Roles of Incentives and Trust. | 9 |
| Rodríguez-Priego, N., & Van Bavel, R | 2016 | The Effect of Warning Messages on Secure Behaviour Online | 10 |
| Rodríguez-Priego, N., van Bavel, R., & Monteleone, S. | 2016 | The disconnection between privacy notices and information disclosure: an online experiment | 10 |
| Rudnicka, A., Cox, A. L. and Gould, S. J. J. | 2019 | Why do you need this? Selective disclosure of data among citizen scientists | 9 |
| Sah, Y. J., & Peng, W | 2015 | Effects of visual and linguistic anthropomorphic cues on social perception, self-awareness, and information disclosure in a health website | 9 |
| Samat, S., Acquisti, A., Clara, S., & Acquisti, A. | 2017 | Format vs. Content: The Impact of Risk and Presentation on Disclosure Decisions | 9 |
| Smith, K. H., Méndez Mediavilla, F. A., & White, G. L | 2018 | The Impact of Online Training on Facebook Privacy | 10 |
| Spottswood, E. L., & Hancock, J. T. | 2017 | Should I Share That? Prompting Social Norms That Influence Privacy Behaviors on a Social Networking Site | 10 |
| Steinfeld, N | 2015 | Trading with privacy: The price of personal information | 10 |
| Sundar, S | 2013 | Unlocking the privacy paradox: do cognitive heuristics hold the key? | 10 |
| Tsai, J., Kelley, P., Drielsma, P., Cranor, L., Hong, J., & Sadeh, N | 2009 | Who’s Viewed You? The Impact of Feedback in a mobile location Sharing System | 10 |
| Tschersich, M | 2015 | Comparing the configuration of privacy settings on social network sites based on different default options | 10 |
| Vitale, J., Tonkin, M.,Ojha, S., Williams, M.-A.. | 2018 | Be More Transparent and Users Will Like You: A Robot Privacy and User Experience Design Experiment | 10 |
| Wang, J., Wang, N., & Jin, H | 2016 | Context Matters? How Adding the Obfuscation Option Affects End Users’ Data Disclosure Decisions | 10 |
| Wang, N., Zhang, B., Liu, B., & Jin, H | 2015 | Investigating Effects of Control and Ads Awareness on Android Users’ Privacy Behaviors and Perceptions | 10 |
| Wang, Y., Leon, P. G., Acquisti, A., Cranor, L. F., Forget, A., & Sadeh, N. | 2014 | A field trial of privacy nudges for Facebook | 10 |
| Warberg, L., Acquisti, A. and Sicker, D. | 2019 | Can privacy nudges be tailored to individuals' decision making and personality traits? | 9 |
| Weydert, V., Desmet, P. and Lancelot-Miltgen, C. | 2020 | Convincing consumers to share personal data: double-edged effect of offering money | 9 |
| Xie, E., Teo, H. H., & Wan, W | 2006 | Volunteering personal information on the internet: Effects of reputation, privacy notices, and rewards on online consumer behavior | 9 |
| Zhang, B., & Xu, H | 2016 | Privacy Nudges for Mobile Applications: Effects on the Creepiness Emotion and Privacy Attitudes | 10 |
| Zhang, B., Wu, M., Kang, H., Go, E., & Sundar, S. S | 2014 | Effects of security warnings and instant gratification cues on attitudes toward mobile websites | 10 |
| Zhu, F., Carpenter, S., & Kulkarni, A | 2012 | Understanding identity exposure in pervasive computing environments | 10 |

**Table A2. Intervention, outcome measure and experimental design of included studies**

| **Author** | **Year** | **Intervention Modality** | **Outcome Measure** | **Experiment Design** |
| --- | --- | --- | --- | --- |
| Acquisti, A., John, L. K., & Loewenstein, G. | 2012 | feedback on other's admissions, presentation - intrusiveness order (social cues and order) | divulging behavior (propensity to respond affirmatively) | survey experiment |
| Adjerid, I., Acquisti, A., Brandimarte, L., & Loewenstein, G | 2013 | reference dependence & framing, and salience of privacy notices - language about protection | propensity to answer personal questions | 2 survey-based experiments |
| Adjerid, I., Peer, E., & Acquisti, A | 2018 | normative and behavioural factors: objective and relative changes in (levels) of privacy protection | likelihood of disclose and actual disclosure | experiment |
| Aiken, K. D., & Boush, D. M | 2006 | trust signals | willingness to provide personal information | between-subjects experiment |
| Alemany, J., del Val, E., Alberola, J. & García-Fornes, A | 2019 | picture nudge, number nudge | Posting behavior | lab experiment |
| Babula, E., Mrzygłód, U., & Poszewiecki, A | 2017 | priming | willingness to disclose private data | lab experiment |
| Baek, Y. M., Bae, Y., Jeong, I., Kim, E., & Rhee, J. W | 2014 | framing of consent forms (opt in/opt out) | choose to protect personal information, | survey experiment |
| Balebako, R., Péer, E., Brandimarte, L., Cranor, L., & Acquisti, A | 2013 | cognitive disfluency (hard to read font) | self-disclosure | survey experiment |
| Becker, M., Matt, C. & Hess, T. | 2020 | persuasive message: attribute framing, argument strength | intention to disclose personal health information | experiment |
| Ben-shahar, O., & Chilton, A | 2016 | simplification of privacy disclosures, format of privacy disclosures | willingness to share personal information | survey experiment |
| Betzing, J. H., Tietz, M., vom Brocke, J. & Becker, J. | 2020 | Transparency in permission requests | accept permission request | experiment |
| Bhatia, J., Breaux, T. D., Reidenberg, J. R., & Norton, T. B | 2016 | vagueness in statement, risk likelihood (privacy violation) | willingness to share personal information | factorial vignettes |
| Brandimarte, L., Acquisti, A., & Loewenstein, G | 2013 | control (release of information and access to information) | willingness to disclose sensitive information | between-subjects experiment |
| Carpenter, S., Shreeves, M., Brown, P., Zhu, F., & Zeng, M | 2018 | warnings | information disclosure | experiment - manipulations |
| Carpenter, S., Zhu, F., & Kolimi, S | 2014 | warnings | identity information disclosure | experiment |
| Carpenter, S., Zhu, F., Zeng, M., & Shreeves, M | 2017 | warnings with sources | disclosure of private information | experiment |
| Chang, D., Krupka, E. L., Adar, E., & Acquisti, A | 2016 | norm shaping design patterns | information divulging behavior | experiment |
| Craciun, G. | 2018 | choice defaults, social consensus | online information sharing | between-subjects experiment |
| Eling, N., Rasthofer, S., Kolhagen, M., Bodden, E., & Buxmann, P | 2016 | coarse- and fine-grained requests | information disclosure | market experiment |
| Feri, F., Giannetti, C., & Jentzsch, N | 2016 | breach notifications | propensity to provide personal sensitive information | experiment |
| Frey, R. M., Bühler, P., Gerdes, A., Hardjono, T., Fuchs, K. L., & Ilic, A | 2017 | standard privacy policy, customer empowerment, blockchain supported system, monetization | willingness to share personal information | experiment |
| Gabisch, J. A., & Milne, G. R. | 2013 | safety cues and rewards | willingness to disclose personal information | experiment |
| Gerlach, J., Widjaja, T., & Buxmann, P. | 2015 | permissiveness of privacy policies | willingness to provide information | vignettes |
| Halevi, T., Kuppusamy, T. K., Caiazzo, M., & Memon, N | 2015 | financial incentive | willingness to share biometric information | experiment |
| Hanson, J., Wei, M., Veys, S., Kugler, M., Strahilevitz, L. & Ur, B. | 2020 | hyper personalised ad in robotext or banner | Information disclosure | lab and online experiments |
| Huang, N., Hong, Y., Chen, P.-Y., & Wu, S.-Y. | 2018 | nudging messages: simple request, monetary incentive, relational capital & cognitive capital (framing) | online social sharing behavior | randomised field experiment |
| Hughes-roberts, T. | 2015 | privacy salient information | information disclosure | experiment |
| Hui, K., Teo, H., & Lee, S | 2007 | privacy assurance (privacy statements, and privacy seals) monetary incentives &information request | consumer disclosure of personal information (actual) | field experiment |
| Hutton, L., Henderson, T., & Kapadia, A. | 2014 | monetary incentives, feedback | sharing of location services (check in) | user study |
| Ilany Tzur, N., Zalmanson, L., & Oestreicher-Singer, G | 2016 | calls to action | information revelation behavior | controlled experiment |
| John, L. K., Acquisti, A., & Loewenstein, G. | 2011 | contextual cues (design features of website) | affirmative admission rate | between-subjects experiment |
| Junger, M., Montoya, L., & Overink, F. J. | 2017 | priming and warning leaflet | information disclosure | survey experiment |
| Keith, M. J., Fredericksen, J. T., Reeves, K. S., & Babb, J | 2018 | video privacy policies ﻿ | information disclosure | field experiment |
| Kim, J., Gambino, A., Sundar, S., Rosson, M., Aritajati, C., Ge, J., & Fanning, C. | 2018 | visual cues, community frame | disclosure of sensitive information | factorial experiment |
| Knijnenburg, B. P., & Kobsa, A | 2013 | type of disclosure justification messages, order of requests | information disclosure decision | experiment |
| Knijnenburg, B. P., Kobsa, A., & Jin, H | 2013 | auto completion tools: auto (traditional), remove & add (alternatives) | information disclosure to external website | experiment |
| Knijnenburg, B. P., Kobsa, A., & Jin, H. | 2013 | fine grained & coarse-grained options | location sharing | experiment |
| Knijnenburg, B., & Kobsa, A | 2016 | granularity of categories, presentation order, defaults, exceptions | sharing tendency | between-subjects experiment |
| Krol, K., & Preibusch, S | 2016 | warning dialogues | information disclosure | between-subjects experiment |
| Kroschke, M., & Steiner, M | 2017 | reviews, peers' behavior=social cues | information disclosure intention | between-subjects experiment |
| Lai, Y.-L., & Hui, K.-L | 2006 | choice frame and defaults (opt in, opt out) | consumer participation in online activities | experiment |
| Larose, R., & Rifon, N. | 2007 | warning labels and privacy seals | personal information disclosure intention | between-subjects experiment |
| Lee, D., Larose, R. | 2011 | personalized social cues: immediacy in the website (design features and speech) | information disclosure intention | experiment |
| Li, H., Sarathy, R., & Xu, H | 2010 | monetary rewards | behavioural intention | experiment |
| Lu, Y., Ou, C. X. J., & Angelopoulos, S | 2018 | monetary incentives or simple reminder | self-disclosure of private information | randomised field experiment |
| Mamonov, S., & Benbunan-Fich, R | 2018 | information security threats (news stories) | non-disclosure of sensitive information | between-subjects experiment |
| Marreiros, H., Tonin, M., Vlassopoulos, M., & Schraefel, M. C | 2017 | privacy messages (news extract/information about positive or negative privacy practice) | propensity to disclose personal information | between-subjects experiment |
| Meier, Y., Schäwel, J., Kyewski, E. & Krämer, N. C. | 2020 | fear appeals (warning), social norms | Self-disclosure | experiment |
| Mettler, T., & Winter, R. | 2016 | social design features, incentives | attitude towards information sharing | design experiment |
| Molina, M. D., Shyam Sundar, S. & Gambino, A. | 2019 | terms and conditions | Information disclosure | factorial experiment |
| Monteleone, S., Bavel, R. Van, Rodríguez-Priego, N., & Esposito, G | 2015 | visceral notices | passive and direct information disclosure | experiment |
| Mothersbaugh, D. L., Foxx, W. K., Beatty, S. E., & Wang, S | 2012 | perceived customization benefits (website customization & frequency of use), level of information control - customisation options | willingness to disclose information | online experiment |
| Mukherjee, S., Manjaly, J. A., & Nargundkar, M | 2013 | monetary cues | willingness to disclose personal information | experiments |
| Nosko, A., Wood, E., Kenney, M., Archer, K., De Pasquale, D., Molema, S., & Zivcakova, L. | 2012 | priming story | inclination towards information disclosure | experiment |
| Peer, E., & Acquisti, A | 2016 | reversibility cue | self-disclosure depth | experiment |
| Preibusch, S., Krol, K., & Beresford, A. R | 2013 | mandatory fields, compensation | voluntary data disclosure | field experiment |
| Premazzi, K., Castaldo, S., Grosso, M., Raman, P., Brudvig, S., & Hofacker, C. F. | 2010 | compensation of different types, trust (excerpt) | willingness to divulge information, behavioural information disclosure, behavioural disclosure of sensitive information | between-subjects experiment |
| Rodríguez-Priego, N., & Van Bavel, R | 2016 | design of security messages | information disclosure (sign up) | experiment |
| Rodríguez-Priego, N., van Bavel, R., & Monteleone, S. | 2016 | design of search engine | disclosure of personal information | experiment |
| Rudnicka, A., Cox, A. L. & Gould, S. J. J. | 2019 | motivational message | Disclosure of sensitive information | experiment |
| Sah, Y. J., & Peng, W | 2015 | visual and linguistic anthropomorphic cues | non-disclosure of personal information | –between-subjects experiment |
| Samat, S., Acquisti, A., Clara, S., & Acquisti, A. | 2017 | privacy notices | willingness to disclose personal information | survey experiment |
| Smith, K. H., Méndez Mediavilla, F. A., & White, G. L | 2018 | Facebook privacy training | intention to disclose personal information | quasi experiment |
| Spottswood, E. L., & Hancock, J. T. | 2017 | visual cues | disclosure frequency | experiment |
| Steinfeld, N | 2015 | monetary rewards | willingness to grant access to Facebook profile | survey experiment |
| Sundar, S | 2013 | benefit heuristic priming, fuzzy boundary heuristic priming (video clips), personalisation cues (generic website & personalised website) video and website | information disclosure | between-subjects experiment |
| Tsai, J., Kelley, P., Drielsma, P., Cranor, L., Hong, J., & Sadeh, N | 2009 | feedback | willingness to share location | field experiment |
| Tschersich, M | 2015 | default privacy settings | individual privacy configuration behavior | experiment |
| Vitale, J., Tonkin, M.,Ojha, S., Williams, M.-A.. | 2018 | embodied robot or on a disembodied kiosk, transparency | willingness to provide personal information (face enrolment, fb request) | between-subjects lab experiment |
| Wang, J., Wang, N., & Jin, H | 2016 | data obfuscation options | user tendency to release data | experiment |
| Wang, N., Zhang, B., Liu, B., & Jin, H | 2015 | privacy notice dialogs | information disclosure | factorial between-subjects experiment |
| Wang, Y., Leon, P. G., Acquisti, A., Cranor, L. F., Forget, A., & Sadeh, N. | 2014 | visual cues and time delays | online information disclosure | field trial |
| Warberg, L., Acquisti, A. & Sicker, D. | 2019 | opt in, opt out, social norms, framing | Information disclosure | experiments |
| Weydert, V., Desmet, P. & Lancelot-Miltgen, C. | 2020 | monetary compensation, control over data | Willingness to share data | survey experiment |
| Xie, E., Teo, H. H., & Wan, W | 2006 | privacy notices, rewards, reputation | willingness to provide personal identifiable, willingness to provide demographic information | factorial vignettes |
| Zhang, B., & Xu, H | 2016 | frequency and social nudges | disclosure comfort | –between-subjects experiment |
| Zhang, B., Wu, M., Kang, H., Go, E., & Sundar, S. S | 2014 | security warnings and instant gratification cues | social media disclosure | factorial between-subjects experiment |
| Zhu, F., Carpenter, S., & Kulkarni, A | 2012 | RationalExposure model - interface | information disclosure | lab experiment |

**Meta-analysis results excluding incentives**

To identify potential changes in the results if papers with incentives were excluded, the meta-analysis was run without them. The overall effect size without papers involving incentives is [95% confidence interval] = 0.31 [0.24, 0.39]; Test for overall effect: Z = 8.32 (P < 0.00001); Heterogeneity: Tau² = 0.12; Chi² = 1057.28, df = 112 (P < 0.00001); I² = 89; Test for subgroup differences: Chi² = 5.52, df = 2 (P = 0.06), I² = 63.8%. Furthermore, for the strategies increasing disclosure: Hedges' g = 0.32 [0.23, 0.41], Heterogeneity: Tau² = 0.07; Chi² = 307.53, df = 45 (P < 0.00001); I² = 85%, Test for overall effect: Z = 7.03 (P < 0.00001). No change was found for nudging strategies to decrease disclosure, as all papers with incentives had manipulations intended to increase disclosure. Test for subgroup differences (increase vs. decrease): Chi² = 4.35, df = 1 (P = 0.04), I² = 77.0%.

Overall, it becomes evident that there are no significant changes to the results when studies with incentives are excluded, and thus no changes to the conclusions of the study.
