# Supplementary material for: Privacy nudges for disclosure of personal information: A systematic literature review and meta-analysis
Source: PLoS One. 2021 Aug 27;16(8):e0256822. doi: 10.1371/journal.pone.0256822 (PMC8396794; doi:10.1371/journal.pone.0256822)
Supplement: S1 Protocol — (PDF) [file pone.0256822.s003.pdf]

Privacy Nudges for Disclosure of Personal Information: A Systematic Review Protocol

Athina Ioannou, Iis Tussyadiah, and Graham Miller
School of Hospitality and Tourism Management
University of Surrey, United Kingdom
{a.ioannou; i.tussyadiah; g.miller}@surrey.ac.uk

Mario Weick
Department of Psychology
Durham University, United Kingdom
mario.weick@durham.ac.uk

Shujun Li
School of Computing
University of Kent, United Kingdom
S.J.Li@kent.ac.uk

2 November 2018

1.0 Background

Human decision making is inherently complex and imperfect. Immersed in digital environments and while performing online activities, individuals are faced daily with numerous privacy and security decisions: configuring visibility in social networking sites, allowing access to sensitive data in mobile apps, clicking or ignoring links embedded in emails, etc. Various factors such as heuristics, cognitive and behavioural biases, and incomplete and asymmetric information can affect privacy decision and behaviour, often leading to deficient and regrettable choices.

Studies have suggested the use of paternalistic interventions or nudges to guide and assist people into making more beneficial and less regrettable security and privacy decisions. A nudge is “any aspect of the choice architecture that alters people’s behaviour in a predictable way without forbidding any options or significantly changing their economic incentives” (Thaler and Sunstein, 2008, p.6). Nudging in a digital environment includes “the use of user interface design elements to guide people’s behaviour in digital choice environments” (Schneider and Weinmann, 2018, p. 68). Aiming to improve users’ well-being, nudging can influence decision making without forcing users and safeguarding them freedom of choice. Existing research on nudging has been conducted mostly in offline environments (Schneider and Weinmann, 2018). However, there is a growing body of literature on the use of digital nudging on individual privacy behaviour and decisions.

This systematic review aims to provide a comprehensive overview of the existing empirical research on nudging and its effects on privacy and security decisions. More specifically, this review will appraise empirical studies examining the effects of nudging strategies on disclosure of personal information as a specific privacy decision. Information disclosure (also known as *data disclosure* or *online disclosure*) refers to the act of making information accessible to other interested parties. In the current review, we are interested in information disclosure of personal information of a user during an online activity. According to Chapter 1, Article 4 of the GDPR (Regulation 2016/679, 2016), personal data is any information that can identify an individual, such as biographical information, workplace data and education information, location data, physical, physiological, genetic, mental, economic, cultural or social data of a person.

2.0 Objective

The objective of this systematic review is to investigate the effects of various intervention (nudging) strategies on disclosure of personal information.

3.0 PICO Framework

For the purposes of this review, the population, intervention, and outcome(s) to inform the review objectives are presented below:

Population	Intervention	Comparison	Outcome
Individuals using technology (users)	Nudging (intervention) strategies, including but not limited to nudging with information, presentation, default, timing, incentive, etc.	n/a	Information disclosure (sharing of personal data)

4.0 Evidence Gathering and Study Selection

4.1 Database Searching

The following databases will be searched with a pre-determined search strategy: Scopus, Google Scholar, ACM Digital Library, Web of Science, and Science Direct. In cases where the search results are small in number, search terms will be reduced to maximize the search sensitivity. In case where search results are very large, filters will be applied. Disciplines of interest are mainly around, but not limited to information systems (IS) and human-computer interaction (HCI). Disciplines of interest also include behavioural economics, computer science, (business) management, and other relevant areas.

4.1.1 Search Strategy

The following table presents the search strategy for this systematic review:

Search Strategy	#1 AND #2 AND #3 AND #4 AND #5
Concepts	Keywords
#1 Privacy	“privacy” OR “confidential*” OR “security”
#2 Personal Information	“personal information” OR “personal data” OR “sensitive information” OR “sensitive data” OR personal information OR personal data OR sensitive personal information OR private information OR private data
#3 Information Disclosure	“information disclosure” OR “willingness to disclose” OR “intention to disclose” OR “likelihood to disclose” OR “willingness to share” OR “intention to share” OR “data sharing” OR “likelihood to share” OR “disclosure behavio*” OR “data disclosure” OR “online disclosure” OR shar* OR “self-disclosure” OR “online information sharing”
#4 Nudging	“nudge*” OR “nudging” OR “intervention*” OR “experiment*” OR “paternalis*” OR “prod” OR “randomi* control trial” OR “quasi-experiment” **AND/OR** “choice architecture” OR “default” OR “framing” OR “priming” OR “incentive*” OR “monet*”
#5 Online	“online” OR “internet” OR “web” OR “digital” OR “software”

4.1.2 Other Sources

Grey Literature, such as industrial or governmental reports, will also be considered.

4.1.3 Reference Searching

Bibliographies of those papers that match the eligibility criteria below will be searched by hand to identify any further, relevant references, which will be subject to the same screening and selection process.

4.2 Eligibility Criteria

After gathering the evidence, the following eligibility criteria will be applied to the results. All identified references will be screened using a three-stage approach to review the title, abstract, and full text as follow:

- Title screening will be performed by one researcher and checked by another researcher for consistency.
- Abstract reading will be performed by two researchers and checked for consistency.
- Full-text reading will be performed by two researchers and checked for consistency.

Where apparent discrepancy occurs, agreement will be reached either by consensus or by including a third, independent researcher.

4.2.1 Types of studies

All empirical studies are eligible for inclusion, including grey literature. Empirical studies reporting experimental manipulations and quasi-experimental variations are considered eligible. Studies conducted in the laboratory, field, and online are eligible.

4.2.2 Types of participants

This review focuses on studies with participants using technology, such as Internet, social media, e-commerce websites, mobile phones, and other digital platforms. There are no age restrictions. Where information is available on individuals' health status, we will focus on studies with healthy participants.

4.2.3 Types of interventions

The review focuses on studies using one or more of the following intervention strategies (nudges) as mentioned in the work of Acquisti et al. (2017): information (feedback and education), presentation (framing, ordering, salience and structure), default, timing, and incentive (priming).

4.2.4 Outcome measures

Studies of interest are considered those that depict the intervention strategies as independent variable(s) and intention/willingness to disclose or share personal information as well as actual disclosure or sharing behaviour as dependent variable(s). Studies that included antecedents of dependent and independent variable(s) as well as mediators and/or moderators in this relationship are also considered eligible.

4.2.5 Exclusion criteria

Papers published in languages other than English are excluded. During the last decade, the privacy of individuals has been affected by new technological solutions such as smartphones and Internet of things. As a consequence, studies published before 2006 are excluded because the meaning of privacy might have changed in the years following 2006 (Gerber, Gerber and Volkamer, 2018). Also, theoretical papers, studies conducted in clinical settings or with clinical samples (e.g. visually impaired) or special populations (e.g. abuse survivors), as well as studies investigating the effect of nudging interventions on outcomes other than information disclosure, such as password creation, selection of secure Wi-Fi, intention towards policy compliance, intention to install application etc., are excluded. Failure to meet any one of the above eligibility criteria will result in exclusion from the review and any apparent discrepancies during the selection process will be resolved through discussion or, in case no agreement is reached, through consultation of an independent reviewer. The number of excluded studies, including reasons for exclusion for those excluded following review of the full text, will be recorded at each stage.

5.0 Data Extraction

Following the initial selection of literature, information will be extracted from relevant papers in the following way. Information to be extracted includes: full reference (including name of author(s), year of publication, and publication), description of the intervention strategy, dependent variable(s) (e.g. intention to disclose information or actual disclosure behaviour), antecedent, moderator, and mediator variables(s), and effects of intervention on dependent variable(s). We will record the direction and significance of any reported effects, and where possible effect size estimates. Where studies are excluded, reasons for exclusion will be recorded. The final decision for inclusion or exclusion will be made by a team consisting of the researchers conducting the review. Any potential disagreement will be resolved through discussion.

6.0 Data Synthesis

The availability of appropriate data and resources will determine the precise nature of the data aggregation method. If possible, we endeavour to conduct a meta-analysis.

7.0 Dissemination

A manuscript will be prepared for submission to a peer-reviewed journal.

References

Acquisti, A. *et al.* (2017) 'Nudges for Privacy and Security: Understanding and Assisting Users' Choices Online', *ACM Computing Surveys*, 50(3). doi: 10.2139/ssrn.2859227.

Regulation 2016/679 of 27th April 2016 on the protection of natural persons with regard to the processing of personal data and on the free movement of such data, and repealing Directive 95/46/EC (General Data Protection Regulation) (2016), *Official Journal of the European Communities*, pp. 1–88. doi: http://eur-lex.europa.eu/pri/en/oj/dat/2003/l_285/l_28520031101en00330037.pdf.

Gerber, N., Gerber, P. and Volkamer, M. (2018) 'Explaining the privacy paradox: A systematic review of literature investigating privacy attitude and behavior', *Computers and Security*. Elsevier Ltd, 77, pp. 226–261. doi: 10.1016/j.cose.2018.04.002.

Schneider, B. Y. C. and Weinmann, M. (2018) 'Digital Nudging : Guiding Online User Choices through Interface Design', *Communications of the ACM*, 61(7), pp. 67–73. doi: 10.1145/3213765.

Acknowledgement

This work was part of the "PRIvacy-aware Personal Data Management and Value Enhancement for Leisure Travellers" (PriVELT) Project, supported by the UK's Engineering and Physical Sciences Research Council (EPSRC), in collaboration with University of Kent, University of Warwick and University of Durham (Grants: EP/R033196/1, EP/R033749/1, and EP/R033609/1).
