# Supplementary material for: Privacy nudges for disclosure of personal information: A systematic literature review and meta-analysis
Source: PLoS One. 2021 Aug 27;16(8):e0256822. doi: 10.1371/journal.pone.0256822 (PMC8396794; doi:10.1371/journal.pone.0256822)
Supplement: Attachment — Submitted filename: PLOS_Responseletter.docx [file pone.0256822.s004.docx]

| Reviewer #1 |  |
| --- | --- |
| This paper presents a meta-analysis of literature on digital nudging. It is well written, coherent, clear, and well structured. It steers clear of the question regarding the overall effectiveness of digital nudging, as this would require knowing about the studies with a null result that were never published. In other words, the paper shows an awareness of publication bias in experimental studies. The paper therefore limits itself to an analysis the corpus of studies that it collected using clear and consistent criteria. I enjoyed reading it and I believe it can make an important contribution to the literature on digital nudging. | We thank the Reviewer for their constructive feedback. We have revised the manuscript addressing the questions and recommendations from both reviewers. |
| General comments  I have one major concern. The authors rely on Acquisti et al.’s (2017) taxonomy of nudges, and group their studies according to the categories of information, presentation, defaults, and incentives. They neglect reversibility (error resiliency) and timing, which they should acknowledge and possibly explain.  Peer and Acquisti, 2016, for example, included in the meta-analysis, would seem to fit under reversibility.  However, the main problem lies with the category ‘incentives’. The whole point of nudging is to go beyond ‘traditional’ policy approaches to influencing behaviour, like bans or taxes. To influence behaviour by offering an incentive is not behavioural economics at all, it is simply economics. It is the most basic, traditional economic approach to influencing behaviour. This is why the demand curve slopes downward and the supply curve slopes upward: because price matters. A monetary incentive, therefore, should not be considered a nudge. The authors acknowledge this when they quote Thaler and Sunstein’s (2008) definition of a nudge on page 3: a nudge is ‘any aspect of the choice architecture that alters people’s behavior in a predictable way without forbidding any options or significantly changing their economic incentives.’ I realise the authors are relying on an existing taxonomy, namely Acquisti et al.’s. But it seems to me, from a cursory glance, that Acquisti et al. had a more nuanced view of incentives. In some cases, rewards were non-monetary. In others, they intended to offset existing biases, such as hyperbolic discounting. Still, in other cases, biases such as loss aversion were leveraged to shape the perception of rewards and punishments. I would invite the authors to reflect on this problem. If the use of the incentive category is justified (à la Acquisti et al.), they should explain this. If it is not, the incentive category and the four papers within it should be excluded. | Thank you very much for your comments and constructive feedback. We have revised the manuscript adding justification about the dimensions of reversibility and timing. No papers were identified to be in these categories, thus this manuscript focused on the main four categories from the framework. For the paper of Peer and Acquisti (2016), it fits both reversibility and presentation nudging. According to Acquisti et al. 2017, the dimensions are not mutually exclusive. Thus, for purposes of comparability, we classified the paper under the presentation nudging.  Regarding the category ‘incentives’, we agree that the definition of Thaler and Sunstein (2008) mention the economic incentive aspect. However, we are following Acquisti et al. (2007)’s framework in this review; the authors specifically cite previous research using monetary incentives as a nudging strategy.  [see, e.g.,Christin, Egelman, Vidas, and Grossklags (2012). It’s all about the Benjamins: An empirical study on incentivizing users to ignore security advice. In Proceedings of the Conference on Financial Cryptography and Data Security (FC’12). Springer-Verlag, Berlin, 16–30].  Furthermore, we have revised the meta-analysis part of the manuscript to report results with the exclusion of papers using incentives (see S1 in Appendix), showing that there are no significant differences in the results and no changes to the study conclusions with the exclusion of incentives. |
| The paper is a bit sloppy when quoting references. For one, it seems to have been written with a different referencing system in mind (Harvard or APA), which sometimes leads to inelegant phrasing, e.g. ‘[8] sought to nudge users…’ Acquisti et al. (2017) appears in the text without its reference number. On pages 21 and 22, references 38 and 65 are mentioned twice. The paper would benefit from a proofreading throughout to address these issues. | Thank you very much for your comment. We have revised manually all the references, as some errors had occurred from the referencing platform. |
| On page 11, the paper claims ‘the investigation of privacy nudges has grown significantly during the last decade, since 2013’. Strictly speaking, given the time lag in scientific publications, the growth in investigation probably began in 2011 or 2012. | Thank you for your comment. We have revised this part as per your suggestion. |
| In Section 3.3.4, first par: ‘Research has shown that the higher the amount of the monetary incentive, the higher the percentage of people who shared their information [81].’ Monetary incentive for what? For disclosing or not disclosing? Common sense would dictate that it is for disclosing, but this should be clarified. | Thank you for your comment. We have revised this sentence to show the motivation behind using incentives to influence disclosure. |
| On page 31, the paper claims ‘results of the meta-analysis show that in absolute terms incentives had the strongest effect on users’ information disclosure’, which is not surprising, as incentives work on a different level (as I noted above). I wonder if this strong effect pushes the overall effect of the studies over a certain threshold. In other words, what would be the overall effect of the studies if we excluded incentives? Given that incentives are not really nudges, it would be good to know this. Would conclusion no (2) ‘a meta-analysis of an adequate papers revealed that nudging strategies can effectively influence information disclosure’ still hold? | Thank you for your comment. We have conducted a separate meta-analysis by excluding papers with incentives. The overall effect size without papers with incentives is [95% confidence interval] = 0.31 [0.24, 0.39]; Test for overall effect: Z = 8.32 (P < 0.00001); For reference: Heterogeneity: Tau² = 0.12; Chi² = 1057.28, df = 112 (P < 0.00001); I² = 89;  Test for subgroup differences: Chi² = 5.52, df = 2 (P = 0.06), I² = 63.8%. Therefore, there are no significant changes to the results when studies with incentives are excluded, and thus no changes to the conclusions of the study. More results are available in S1 in Appendix. |
| On page 32, the paper claims that the number of studies in the ‘incentive’ category were five, but elsewhere it says they were four. I guess it means four studies, but five effects. Could you please clarify? | In the paper we denote with *n* the number of papers, with *i* the number of studies (i.e., one paper can have more than one study) and with *k* the number of effect sizes. We have included a footnote on pg.26 to make this clearer. |
|  |  |

| Reviewer #2 |  |
| --- | --- |
| This is an interesting study. You need to some work on the paper if you want to get it published | We thank the Reviewer for their constructive feedback. We have revised the manuscript addressing the questions and recommendations from both reviewers. |
| (1) People do not make imperfect decisions - to them, the decisions are perfect. They make unwise decisions | Thank you for your comment. We have revised the sentence to incorporate your suggestion. |
| (2) You have a habit of not using the author name. e.g. [1] says .... Respect the author enough to name them | Thank you for your comment. This was an error from the referencing system. We have manually revised all references in the manuscript. |
| (3) When a nudge encourages unwise disclosure, it is no longer a nudge which must always be for the good of the nudgee. What it becomes is SLUDGE | Thank you for your comment. We have added a footnote in the manuscript to highlight this aspect. |
| (4) You are conflating privacy and security. Malware is a security problem. Disclosure is a privacy issue. If malware makes data inaccessible, that is an availability issue (from the CIA principles) and thus security | Thank you for your comment. We have revised this part to avoid confusion between privacy with security. |
| (5) when quoting, provide page numbers | Thank you for your comment. This was an error from the referencing system. We have manually added pages on references when needed. |
| 6) Personal information should include mention of telephone numbers and email addresses | Thank you very much for your comment. We have revised this part of the manuscript on pg.4. |
| (7) sec 3.4 referencing problem in first line | Thank you for your comment. We have revised this and corrected the referencing. |
| 8) p26 - last para - what does 84 refer to? | Thank you for your comment. 84 is the total number of nudges. We have revised the sentence to make this clearer. |
| (9) p31 - again, when a nudge is not doing good to the nudgee, it is sludge | Thank you for your comment. We have added a footnote in the manuscript to highlight this. |
| (10) provide a reference for incentives. BTW - nudges, per definition, exclude incentives (rewards) or sanctions. Strictly speaking, anything using an incentive is not a nudge | We have included a reference on pg.33 for the use of incentives. As you didn’t mention a specific page, we hope that this is appropriate. |
| (11) The conclusions and implications section is FAR too long. Call this reflection and tighten it up. The section rambles too much | Thank you for your comment. We have revised this section, making it more structured and concise. |
| (12) add a short and concise conclusion | We have added a short and concise conclusion. |
| (13) at the end you say that more work needs to be done. This is standard in future work sections but dissatisfactory. Say how - you know the literature so say exactly how this should be achieved. | We have revised this section, adding more specific directions for future research. |
| (14) incomplete refs: 2, 13, 15, 24, 35, 29, 48, 95 (is xx correct?), 103 - what is CNBC?, 105 (repeats at end), 46 repeats at end | Thank you for your comment. We have revised the reference list. |
