# Supplementary material for: Privacy nudges for disclosure of personal information: A systematic literature review and meta-analysis
Source: PLoS One. 2021 Aug 27;16(8):e0256822. doi: 10.1371/journal.pone.0256822 (PMC8396794; doi:10.1371/journal.pone.0256822)
Supplement: Attachment — Submitted filename: PLOS_Responseletter_final.docx [file pone.0256822.s005.docx]

**Privacy Nudges for Disclosure of Personal Information**:

A Systematic Literature Review and Meta-Analysis

Responses to Reviewers

| Reviewer #1 |  |
| --- | --- |
| Thank you for taking my comments into account. | We thank the Reviewer for their constructive feedback. We have revised the manuscript addressing the question at hand. |
| Regarding incentives in particular, I am reassured that running the analysis without incentives does not change the main outcome of the paper, which was a big worry. However, I am disappointed that you did not give this issue greater importance in the paper. It is reduced to a footnote and to an analysis in the Supplementary Material. You did not take up my invitation to reflect more on the question of incentives. Just hiding behind Acquisti et al.'s taxonomy as a justification is not enough - in my opinion - to advance scientific knowledge on this issue and move the debate forward. At the very least, you have a glaring contradiction in your paper, which has "Nudges" in its title, cites Thaler and Sunstein's definition that an incentive is *not* a nudge, and then happily embraces Acquisti et al.'s taxonomy without questioning whether the inclusion of incentives is appropriate in this context. I would have expected at least a paragraph where you discuss this issue. You could argue that perhaps it depends on the behaviour being observed. Or you could go into the detail of how Acquisti et al. apply the category of incentives in their study (as I mentioned in my review). Perhaps in the context of privacy an incentive can and should be considered a nudge, in apparent contradiction to Thaler and Sunstein. But the reader would like to know why. I would like to insist on such a reflection on your part. I think it is important, and if we start overlooking these important issues, lying at the core of the behavioural turn in policy-making, the value of the approach will be undermined and it will all start to go downhill from there. Please provide at least a paragraph, possibly in Section 3.3.4, where you address this so that the reader who is interested in nudges and privacy can better understand if - in the context of privacy and following the definition of Thaler and Sunstein - an incentive can or cannot be considered a nudge. | Thank you very much for your comment.  We have revised the manuscript to have three paragraphs in Section 3.3.4, clarifying the issue of the inclusion of incentives as nudges in the paper. We reflect on the disagreement regarding the relation of nudges and incentives in the existing literature. We offer justification of the inclusion of incentives as nudges in the context of privacy.  We also clarify that the present study does not seek to offer theoretical justification on how to distinguish interventions in the contexts of privacy, but rather aims to offer empirical evidence on the effectiveness of various interventions towards influencing privacy decision making. |

| Reviewer #2 |  |
| --- | --- |
| I'm happy with the revisions the authors made. They have been responsive to my comments and the paper is much improved. | We thank the Reviewer for their constructive feedback and comments throughout the review process. |
